# Magnetic-field-dependent stimulated emission from nitrogen-vacancy centers in diamond

**DOI:** 10.1126/sciadv.abn7192

**Published:** 2022-06-03

**Authors:** Felix A. Hahl, Lukas Lindner, Xavier Vidal, Tingpeng Luo, Takeshi Ohshima, Shinobu Onoda, Shuya Ishii, Alexander M. Zaitsev, Marco Capelli, Brant C. Gibson, Andrew D. Greentree, Jan Jeske

**Affiliations:** 1 Fraunhofer-Institut für Angewandte Festkörperphysik (IAF), Tullastrasse 72, 79108 Freiburg, Germany.; 2 National Institutes for Quantum Science and Technology (QST), 1233 Watanuki, Takasaki, Gunma 370-1292, Japan.; 3 College of Staten Island, CUNY, 2800 Victory Blvd., Staten Island, NY 10312, USA.; 4 Gemological Institute of America, 50 W 47th St. #800, New York, NY 10036, USA.; 5 School of Science, RMIT University, Melbourne, VIC 3001, Australia.; 6 ARC Centre of Excellence for Nanoscale BioPhotonics, School of Science, RMIT University, Melbourne, VIC 3001, Australia.

## Abstract

Negatively charged nitrogen-vacancy (NV) centers in diamond are promising magnetic field quantum sensors. Laser threshold magnetometry theory predicts improved NV center ensemble sensitivity via increased signal strength and magnetic field contrast. Here, we experimentally demonstrate laser threshold magnetometry. We use a macroscopic high-finesse laser cavity containing a highly NV-doped and low absorbing diamond gain medium that is pumped at 532 nm and resonantly seeded at 710 nm. This enables a 64% signal power amplification by stimulated emission. We test the magnetic field dependency of the amplification and thus demonstrate magnetic field–dependent stimulated emission from an NV center ensemble. This emission shows an ultrahigh contrast of 33% and a maximum output power in the milliwatt regime. The coherent readout of NV centers pave the way for novel cavity and laser applications of quantum defects and diamond NV magnetic field sensors with substantially improved sensitivity for the health, research, and mining sectors.

## Teaser

Sensing magnetic fields is improved by stimulated emission from nitrogen-vacancy centers.

## INTRODUCTION

Magnetic field quantum sensors are of increasing importance across a diverse range of fields. Particular examples include the monitoring and diagnosis of medical conditions through the examination of neuronal activity (magnetoencephalography) or cardiological signals (magnetocardiography) (*1*–*4*), in the mining industry for geological exploration of magnetic minerals and magnetic anomaly detection (*5*) or in fundamental studies of magnetism (*6*).

Currently, the best magnetic field sensitivities are reached by superconducting quantum interference devices (SQUIDs) and vapor cells, like spin exchange relaxation-free magnetometers reaching sensitivities below $1\;\text{fT}/\sqrt{\text{Hz}}$ (*7*–*9*). However, SQUIDs require cryogenic cooling with liquid helium, and vapor cells can only be operated in zero-field environments and require heating.

The use of negatively charged nitrogen-vacancy (NV) centers for quantum magnetic sensing has caused great attention since it forms a quantum system that can be operated under ambient conditions in the earth’s magnetic field or other background fields, avoiding the need for cryogenic cooling and shielding, such as required for SQUIDs and vapor cells (*7*, *8*, *10*). Present sensitivities of NV center ensembles reach $\text{pT}/\sqrt{\text{Hz}}$ (*11*, *12*), restricted by fluorescence collection efficiency, low contrast, and reduced spin coherence time in highly doped diamond (*13*, *14*). The improvement of the NV ensemble sensitivity to magnetic fields would enable versatile and robust room temperature sensing applications.

The unique optical and spin properties are due to the isolated NV center’s electronic energy levels in the bandgap of diamond, which enable optical excitation, spin initialization, and detection. This allows for sensing temperature, strain and pressure, and electric and magnetic fields with single or ensembles of NV centers (*15*–*17*). The photoluminescence (PL) differs between the “brighter” spin *m_s_* = 0 and “darker” *m_s_* = ±1 state. The contrast for ensembles of NV centers is typically only ∼5% because of the stray signal from other defects and inhomogeneous broadening leading to a decreasing signal-to-noise ratio (*18*) that can only slightly be improved to 9% by photocurrent detection of magnetic resonance (*19*). Current NV magnetometry is realized by collecting the PL of the NV center, both for single and for ensembles of NV centers. To enhance the detection signal, the spontaneous emission that is sent out into the whole spatial angle is partially collected via optical lenses (*12*, *18*, *20*). In addition to limited PL collection efficiency, the theoretical maximal contrast in the low excitation limit due to quenching by spin-mixing of the spin states *m_s_* = 0, ±1 of an ensemble of NV centers considering all four possible NV directions (*21*) is 22% (section S3).

A boost for the sensitivity is promised by laser threshold magnetometry (LTM) (*22*) due to the competition between spontaneous and stimulated emission. This leads to an expected contrast of almost unity at the lasing threshold. In addition, the coherently emitted photons by stimulated emission in an NV laser are directional and lead to much higher signal collection efficiency compared to spontaneous emission. The predicted sensitivity of $1\;\text{fT}/\sqrt{\text{Hz}}$ (*22*) for LTM would enable overcoming the three orders of magnitude between the predicted and state-of-the-art NV-ensemble sensitivities. Further theoretical concepts for LTM using the NV’s infrared transition (*23*), a combination with diamond Raman lasers (*24*) or visible absorption (*25*), have been explored. Stimulated emission from NV centers has been shown (*26*), as well as amplification by stimulated emission in a fiber cavity (*27*). For this reason, a laser out of NV centers is the goal to enable crossing boundaries in NV center sensitivities. However, despite several studies (*24*, *25*, *27*–*33*), very strong stimulated emission signals or magnetic field–dependent coherent readout have not been achieved so far.

Here, we show magnetic field–dependent light amplification by stimulated emission in an ensemble of NV centers in a high-finesse cavity. The NV centers were pumped at 532 nm, and the cavity was seeded at 710 nm. The cavity output at 710 nm increased by 64% when pumped by the green laser. By applying a permanent magnetic field to the NV ensemble with a major transverse component *B_x_*, i.e., perpendicular to the NV direction, we observed a contrast (magnetic field to no-magnetic field) of 33% due to PL quenching via spin-mixing. This exceeds the theoretical maximum achievable for conventional ensemble PL. Furthermore, we show a previously unkown effect that we call induced absorption as an additional loss channel in the diamond medium, which reduces the amplification and the cavity finesse at pump intensities ≥10 kW/cm^2^. The induced absorption prevents the NV centers from self-sustained lasing activity. We show optically detected magnetic resonance (ODMR) with a contrast of 17%, which is higher than the simultaneously detected contrast of the PL reaching 11%. We reach a shot noise–limited DC sensitivity of $(29.1\pm 2.5)\;\text{pT}/\sqrt{\text{Hz}}$ with a coherent laser signal output in the milliwatt range. This demonstrates experimentally the principle of LTM for the first time.

## RESULTS

### High finesse NV-lasing cavity

**Fig. 1.**
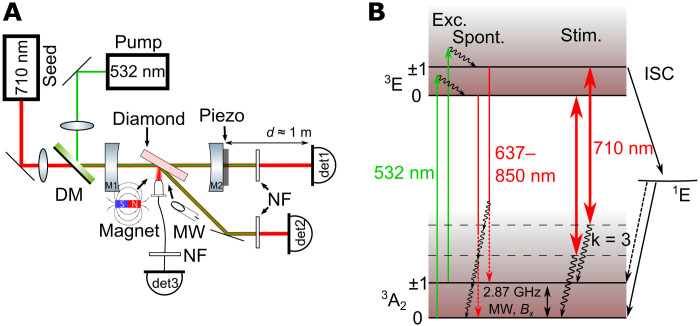
Concept for measuring stimulated emission. (**A**) Experimental setup: The pump laser (532 nm) and the seeding laser (710 nm) are combined with a dichroic mirror (DM) and individually focused into the cavity. The green laser is blocked via 532-nm notch filters (NF). Detected are the transmitted (det1), reflected (det2), and PL signals (det3). (**B**) NV^−^ center energy level schematic: The system is pumped (Exc.) by a 532-nm laser from the ground (^3^A_2_) to the excited state (^3^E). The spontaneous emission (Spont.) spectrum is in the red to near-infrared regime (637 to 850 nm). The effective magnetic field *B_x_*, as well as a continuous microwave drive at 2.87 GHz, lead to spin-mixing. Phonon levels are indicated by the shaded area. The three-phonon transitions of the spin *m_s_* = 0 ± 1 states at λ = 710 nm, where stimulated emission (Stim.) occurs, are indicated with the large double arrow.

The experimental setup in Fig. 1A is designed to study the amplification by stimulated emission and the finesse of the cavity simultaneously. To achieve strong stimulated emission, pumping of a high number of NV centers is needed. For this, we use a highly NV-doped (≈1.8 parts per million) bulk diamond plate of thickness *l* = 295 μm. We pump the NV centers at λ_pump_ = 532 nm with a diode-pumped solid-state laser and a maximum output power of 12 W. A macroscopic pumping volume is achieved by choosing the fundamental TEM_00_ cavity mode to have a beam waist of ω_r_ = 52.0 μm including the diamond. The waist of the fundamental mode leads to a symmetric laser cavity geometry with mirror radius of curvature ROC = 30 mm and geometric cavity length of *L* = 12 mm. To address all NV centers in the mode volume and still maintain a high power density, the beam waist of the green laser ω_g_ = 55.5 μm, measured with a beam camera, is slightly bigger than the cavity mode. The high pump power enables pumping with a maximum power density of 0.1 MW/cm^2^ of the diamond medium mode volume of 0.003 mm^3^ corresponding to ≈10^12^ NV centers.

Figure 1B shows the energy level scheme of the NV center. The maximum gain from NV centers is expected at the emission of the three-phonon sideband at 710 nm (*26*, *29*, *34*, *35*). Additional seeding of the cavity is therefore realized by a laser at λ_seed_ = 710 nm. The amplification experiments show that the losses in the cavity are higher than the gain provided by the NV centers. This prohibits a spontaneous onset of stimulated emission, i.e., self-sustained lasing. Consequently, the system is operated below threshold and requires seeding to detect stimulated emission. To enable pumping and, at the same time, achieve high red intensity to observe stimulated emission, i.e., seeding the cavity, both lasers are combined with a dichroic mirror. Thus, the addressed third phonon-sideband transition (see indicated stimulated transitions in Fig. 1B) corresponds to a four-state laser level system at the peak NV spectrum emission (*35*).

High gain in a laser cavity requires, on the one hand, a high number of emitters. On the other hand, the cavity losses at the lasing wavelength of λ = 710 nm need to be minimized, which leads to a high finesse and consequently many round trips of the emitted photons in the cavity. Thus, the diamond plate is polished on both sides to a surface roughness of *R*_a_ = 0.5 nm and placed in Brewster’s angle to minimize the scattering and reflection losses at the diamond surface, respectively. Furthermore, we achieve an ultralow absorption of the diamond host crystal of μ_abs_ = 0.01 cm^−1^ at λ = 710 nm (Materials and Methods and fig. S4A). To further minimize the transmission losses of the cavity mirrors, we choose two identical, highly reflective mirrors *R* = 99.98%. This results in a poorly impedance-matched cavity; however, we gain an improved ratio of the stimulated emission signal to the signal of the seeding laser transmitted through the cavity.

**Fig. 2.**
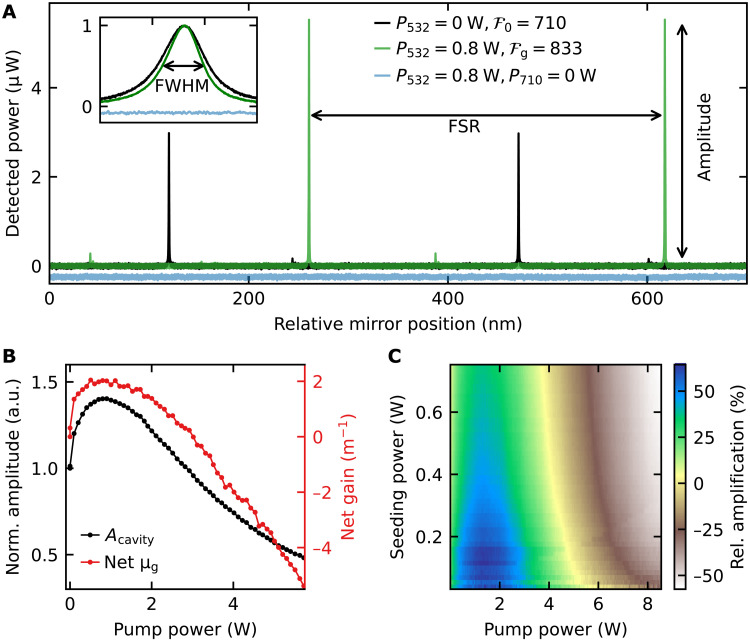
Measurement and amplification of the cavity mode. (**A**) Finesse and amplification measurement of the cavity via scanning the position of one mirror. The black trace shows the signal of the seeding laser only at 710 nm. When the green laser pumps the NV centers, the finesse and amplitude increase (green trace). The signal of only the green laser pumping (blue trace) is shifted for better visibility. The inset shows a zoom into normalized and overlapped peaks. (**B**) Cavity amplitude *A*_cavity_ (black) and net gain μ_g_ (red) as a function of the pump power. *A*_cavity_ is normalized to the amplitude at zero pump power. The net gain is calculated from the measured finesse (section S1). (**C**) Amplification Δ*A* over input pumping power *P*_532_ and input seeding power *P*_710_. The amplification Δ*A* is the relative difference to the case of zero pumping power *P*_532_ = 0 W. Maximal amplification of 64% occurs at 1- to 2-W pump and <0.2-W seeding power. a.u., arbitrary units.

The finesse of the cavity is given by ʿ = FSR/FWHM = Δλ/δλ, where FWHM is the full width at half maximum and FSR is the free spectral range of the resonances (see Fig. 2A). The FSR can be expressed as FSR = *c*/(2*L*_opt_), which is constant when the seeding wavelength is fixed. Here, *c* is the speed of light in vacuum, and *L*_opt_ is the optical cavity length. If we assume a high finesse of ʿ = 800, then it follows that FWHM = 15 MHz. We use the seeding laser at λ_seed_ = 710 nm with a much smaller linewidth (<100 kHz) such that we can measure the FWHM by scanning the thin laser line over the resonance. To precisely measure the finesse of the cavity, the laser is combined with a piezo that linearly scans the second mirror (M2) of the cavity over a maximum range of 3 μm (see Material and Methods).

### Stimulated emission sensing

The NV-diamond–loaded, high-finesse cavity setup allows us to study the amplification behavior when the green laser pumps the NV centers. Figure 2A shows that we achieve a high-finesse cavity of ʿ = 710. In the case of a high finesse, the Airy function can be approximated by a Lorentzian (*36*). When only the red laser is used, the finesse of the passive cavity reads ʿ_p_ = π/(μ_0_*L*), where μ_0_ is the overall loss per unit length of the cavity and *L* is the geometric cavity length. For the active case, i.e., the cavity is seeded by the red laser and the NV centers are pumped and provide gain by stimulated emission, the formula ʿ = π/(μ_0_*L* − μ_g_*l*) (*37*) describes the finesse below lasing threshold. Here, *l* is the geometric length of the diamond gain medium, and μ_g_ is the gain per unit length from the NV centers. The formula shows that the finesse increases because of the gain from the NV centers, i.e., the gain can be seen as “negative loss” (*37*). A change in gain can therefore be observed in the FWHM and finesse of the cavity if the seeding wavelength is constant. It is also distinct from thermal influences on the cavity that would affect the finesse via a change in the FSR. When pumping the NV centers, we see that the additional gain by stimulated emission reduces the loss; consequently, an increase in the finesse is expected (*37*).

The green trace in Fig. 2A shows the detection of the amplification by stimulated emission of the cavity modes. The seeding power was set to *P*_710_ = 7.7 mW. By pumping the NV centers, we detect a clear amplitude amplification of 45% of the resonances, which corresponds to a raise in the cavity output power from 3.0 to 5.5 μW. In addition, the increase of the finesse is caused by the reduction of the FWHM, as shown in the inset of Fig. 2A. When only the green laser is pumping the NV centers, we detect a flat signal. This verifies that the detected amplification is not caused by the spontaneous emission of the NV centers.

Figure 2B shows the behavior of the amplification as a function of the green pump power by detecting the amplitude of the cavity mode *A*_cavity_. The maximum of the amplification is at ≈1 W, corresponding to a pump intensity of 103 W/mm^2^. We investigate the origin of the amplification by the detection of the cavity FSR and FWHM during a scan of the pump power (fig. S1). We find that the amplification comes from a reduction of the FWHM, that is, a change in the net gain of the NV centers. The gain also leads to a higher number of photons in the cavity, as expected from stimulated emission. The FSR is constant when the pump power is increased. This proves that no thermal expansion is influencing the finesse and amplitude, as FSR ∝ *L*_opt_.

To exclude that the increase of the finesse comes from a reduction of the initial absorption of the sample, we investigate samples with an absorption between 0.3 and 0.01 cm^−1^ and find out that the strongest amplification comes from the sample with the lowest absorption (fig. S4A), which we use for all other measurements. Calculations of the NV ground-state three-phonon level population in combination with the absorption and amplification indicate that the initial absorption of the NV center with a wavelength of 710 nm does not play a substantial role (fig. S4B). As the initial absorption of the used sample in our experiments has an ultralow absorption of 0.01 cm^−1^, this shows that we see stimulated emission and not a reduction in initial absorption.

Gain in the cavity is directly detected by the amplification of the amplitude *A*, which is proportional to the detected power and intracavity power. The amplitude, which is the maximum of the cavity resonance, would correspond to the power output of a cavity locked to its maximum. We use the cavity amplitude of the transmitted *A*_trans_ (det1) or reflected *A*_ref_ (det2) signal for further quantification of the cavity behavior.

The net gain from the NV centers is shown Fig. 2B (red trace). It is calculated from the finesse by $\mu_{g}=\pi /l(F_{0}^{-1}-F_{g}^{-1}$); (section S1). Here, ʿ_0_ and ʿ*_g_* are the finesse at zero and nonzero pump power, respectively. The maximal net gain is 2.04 m^−1^ at ≈1 W. For higher pump powers, the gain and the amplitude decrease. At a pump power above 3 W, both curves decrease below their initial value at zero pump power, ending the regime of net gain. The high pumping power regime above 3 W shows a linear increase of the losses (FWHM; fig. S1). Photoionization is only a minor effect and cannot be the main cause of this additional loss (see section S7). An investigation of the time scales of the amplification and induced losses when turning the laser on and off suggest a previously unknown effect of induced absorption at the wavelength of 710 nm in the diamond caused by the green pumping laser (see section S7). This induced absorption was likely a major hurdle in previous attempts at lasing (*25*, *27*, *29*). The data show that this effect of induced absorption compensates the gain by stimulated emission at a pumping power of *P*_532_ ≈ 3 W.

The amplification is dependent on the pump and seeding power as shown in Fig. 2C. Here, the amplification Δ*A* = (*A* − *A*_0_)/*A*_0_ is calculated from the relative difference between the amplified cavity amplitude *A* and the amplitude without pumping *A*_0_. The measurement shows an optimal power regime for the amplification by stimulated emission. The highest amplification by stimulated emission is at *P*_532_ = 1 to 2 W and small red seeding powers *P*_710_ < 0.2 W. In this regime, the amplification by stimulated emission reaches ≈64%.

### Analysis of the magnetic field contrast by stimulated emission in a seeded cavity

The use of stimulated emission enables us to reach a higher contrast that exceeds the contrast reached by conventional PL detection. The higher contrast is due to the different magnetic field dependency of the emission rates for PL and stimulated emission. The PL emission rate of the NV centers is given by Γ_PL_ = ρ_e_*N*_NV_Γ, where ρ_e_*N*_NV_ is the number of NV centers in the excited state and Γ is the inverse excited state lifetime (*21*). The stimulated emission rate Γ_st_ = ρ_e_*N*_NV_*Gn* is proportional to the product of the number of photons in the cavity *n* and the coupling constant *G* between the NV centers and the cavity (*37*).

In the steady state of constant pumping, an externally applied transverse magnetic field *B_x_* reduces the excited state population ρ_e_(*B_x_* ≠ 0) < ρ_e_(*B_x_* = 0) by mixing the spin states *m_s_* = 0, ±1, i.e., the PL emission Γ*_PL_*. The stimulated emission rate reduces in addition to the exited state population ρ_e_(*B_x_* ≠ 0) < ρ_e_(*B_x_* = 0) also by a decreasing number of photons *n*(*B_x_* ≠ 0) < *n*(*B_x_* = 0) due to a reduced emission of the NV centers into the cavity (see section S8). The stimulated emission rate is consequently the product of two quantities reducing with magnetic field. The nonlinearity caused by the lasing cavity enhances the contrast.

**Fig. 3.**
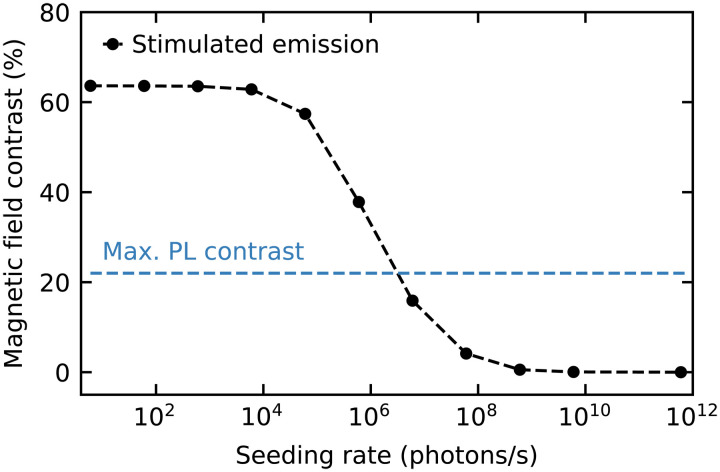
Simulation of the magnetic field contrast by stimulated emission in a seeded cavity. The contrast by stimulated emission is exceeding the PL contrast for low external seeding, i.e., a high contribution of the stimulated emission to the detection signal. The contrast is calculated by the relative difference of the stimulated emission rates *C* = 1 − Γ_st_(*B* ≠ 0)/Γ_st_(*B* = 0). A detailed calculation can be found in section S8.

We calculated a numerical example of a seeded cavity with an NV center (section S8) and provide a direct comparison of contrast achievable with stimulated emission and spontaneous emission in Fig. 3. The calculation shows quantitatively the magnetic field contrast by stimulated emission in a seeded cavity below threshold. As a comparison, the maximal theoretical contrast of 22% by PL detection is indicated as well (section S3). In the regime of a high stimulated emission from the NV centers contributing to the overall signal, i.e., low external seed (<10^6^), the contrast is higher than 22%.

### Magnetic field dependency

After the demonstration of strong light amplification, we investigate the magnetic field dependency of the measured amplification. As only the emission of the NV center decreases with an external magnetic field, this proves that the amplification is caused by the NV centers. We particularly focus on the question whether the competition between stimulated and spontaneous emission, i.e., the nonlinearity of the cavity, leads to a signal amplification, which should show up via increased contrast compared to spontaneous emission as we expect from the analysis in the previous section.

We performed the measurements on the same setup as described in Fig. 1A. To apply a homogeneous magnetic field inside the diamond, we use as a first experiment a strong permanent magnet. The magnet is guided from the top by a linear piezo stage (shown in Fig. 1A from the side for purposes of representation). The magnetic field is applied in the (100) crystal direction. The diamond contains NV centers in all four possible NV directions. In this configuration, the projections of the applied magnetic field onto the perpendicular plane to the NV axis is symmetric and of the same magnitude for all NV orientations. The angle between the NV axis is α = 109.5^∘^ (*21*). The effective magnetic field that leads to a mixing of the spin states is then calculated by *B_x_* = *B* sin (α/2), where *B* is the magnitude of the measured magnetic field. We assume a constant magnetic field within the mode of the diamond of diameter 2ω_0_ ≈ 110 μm. The excited NV centers in the diamond mode in the experiment are located at a distance of (2 ± 1) mm from the magnet. The effective magnetic field is *B_x_* = (146 ± 25) mT (sections S2 and S3).

**Fig. 4.**
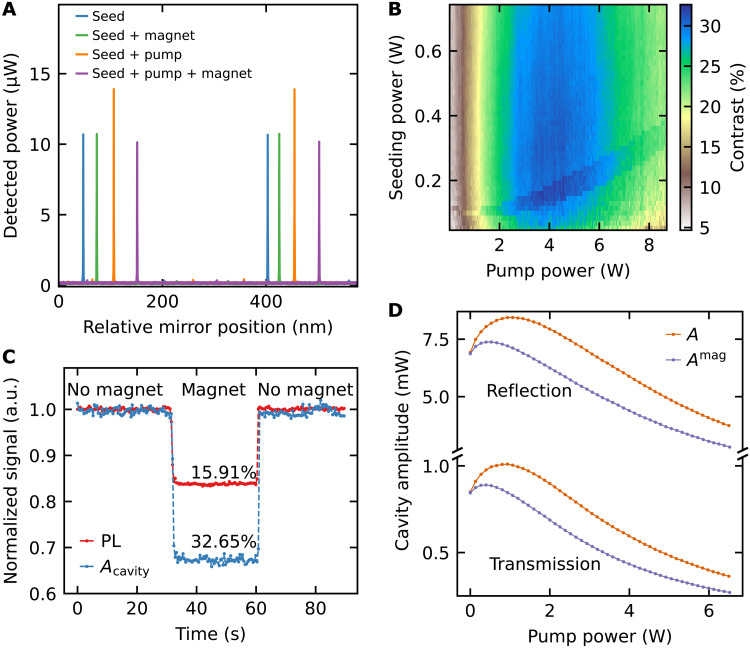
Magnetic field–dependent amplification with ultrahigh contrast. (**A**) Detection of the transmitted cavity power over the relative mirror position. The transmission of only the red laser (Seed) is not influenced when a strong magnet is brought close to the diamond (Seed + magnet). Strong amplification of the mode appears when the green laser pumps the NV centers (Seed + pump). Bringing a magnet close to the diamond substantially reduces the amplified signal (Seed+ pump + magnet). The power for the seed and pump laser is *P*_710_ = 0.3 W and *P*_532_ = 1.37 W, respectively. The data are shifted horizontally for clarity. (**B**) Contrast of the detected amplitude over the pump *P*_532_ and seeding *P*_710_ power. (**C**) Transmitted peak amplitude (det1) of the cavity mode *A*_cavity_ and laterally emitted PL (det3) are detected simultaneously over time. When the magnet is brought close to the diamond for 30 s, both signals decrease. (**D**) Measurement of the reflected (det2) and transmitted (det1) amplitudes in absolute values with (*A*^mag^) and without (*A*) magnet over the pump power *P*_532_ (note the subdivided vertical axis). The seeding power is set to *P*_710_ = 1.14 W. The data represent the mean value of eight measurements.

Figure 4A shows measurements of the cavity TEM_00_ modes under different conditions. The cavity mode excited by the red laser gives a finesse of ʿ = (958 ± 6) and is not influenced by the magnetic field. The coherent stimulated emission when pumping the NV centers couples into the cavity mode and amplifies the amplitude of the resonances. The result leads to an amplification of Δ*A* = 30% and a finesse of ʿ = (1086 ± 2). The presence of a magnetic field suppresses the amplification substantially and leads to a finesse of ʿ = (926 ± 3). The decreasing finesse shows that there is a reduction in gain in the cavity when a magnetic field is applied. The reduction is due to the transverse magnetic field component *B_x_* (see section S3) that leads to mixing of the brighter *m_s_* = 0 and darker *m_s_* = ±1 spin states of the NV center ground state. These darker *m_s_* = ±1 states show an intersystem crossing to the long-living singlet state (^1^*A*), depicted in Fig. 1B. The measurements demonstrate the first magnetic field–dependent amplification by stimulated emission from NV centers.

The reduction of the signal is equal to the entire amplification, suggesting that because of the cavity enhancement, the entire signal from the NV centers is reduced by the magnetic field. This opens the potential to achieve close to 100% contrast when the seeding laser is filtered out, e.g., via lock-in detection where the NV emission is modulated and a lock-in amplifier rejects the unmodulated seeding laser signal. In this work, we analyze contrast only on the basis of the combined signal from NV centers and seeding laser.

Furthermore, we investigate the contrast caused by the magnetic field–dependent stimulated emission. The amplitude of the cavity mode is detected while scanning the pump and seeding power, once with and once without the magnet close to the NV centers. The optimal power regime for the relative detected contrast is shown in Fig. 4B. The contrast is calculated from Contrast = (*A* − *A*^mag^)/*A*, where *A*^mag^ and *A* are the detected amplitude with and without a magnet, respectively. The contrast reaches a maximum value of (32.6 ± 0.4)%. The range above 25% is at pump powers between 2 and 6 W over a large red seeding power regime of 10 to 650 mW.

If we compare the optimal amplification regime in Fig. 2C to the optimal pump power for maximal contrast, then the optimal contrast is at higher pump powers than the optimum for amplification. We explain the difference by the interplay between the amplification by stimulated emission and the induced absorption discussed in the previous section. We have found that the induced absorption is dependent on the pump power, that is, on the excitation rate of the NV centers. The gain μ_g_ by stimulated emission depends on the excitation rate as well, as it is proportional to the excited state population μ_g_ ∝ ρ_e_ (*38*). To optimize the magnetic field dependency, the NV centers have to be spin-polarized to the *m_s_* = 0 ground state. The spin polarization is also dependent on the excitation rate. As the beam waist in the focal spot is *w*_g_ = 55.5 μm, high pump powers are needed for sufficient spin polarization. We see from the measurements that at the point of maximal amplification, which is an interplay between the stimulated emission and induced absorption, the spin polarization is not high enough to reach the highest magnetic field response and contrast. That point is reached at higher excitation rates where the induced absorption is already compensating the stimulated emission.

Final verification of the enhanced magnetic field–dependent response of the NV centers is done by a direct comparison between the magnetic field–dependent PL and coherent cavity output (det3 and det1 in Fig. 1A, respectively). A transverse magnetic field is introduced and removed, which mixes the spin states and reduces the NV signal. We use a pump power *P*_532_ = 3.4 W and seeding power *P*_710_ = 25 mW in the regime of high contrast of Fig. 4B. In Fig. 4C, the NV center PL is detected over time. The measured contrast of the PL reaches 15.91%, which is a high contrast when measuring the PL of NV ensembles. It is reduced because of inhomogeneous broadening and background PL (e.g., from NV^0^) (*18*). The maximal contrast via PL in the weak-excitation limit that can be reached with a permanent magnet is 22% (Fig. 3).

Simultaneously, the amplitude of the cavity resonance is detected in Fig. 4C. The contrast of the cavity mode (*A*_cavity_) is 32.65%, that is, more than twice as high as the contrast detected by collecting only the PL of the NV centers. Our coherent cavity readout surpassed the fundamental contrast that is possible via PL collection. The measurement shows the higher contrast because of the competition between spontaneous and stimulated emission in the cavity. The magnetic field–induced reduction of gain results in a decreased NV center emission that is enhanced by the nonlinear cavity effect.

Figure 4D shows the magnetic field–dependent behavior of the transmitted and reflected signal amplitude over the pump power. The magnetic field influences the transmitted and reflected signal in the same way. The maximum of the amplitude is shifted to lower pump powers in the presence of a magnetic field compared to no magnetic field, and a reduction of the amplitude with magnetic field over the complete pump power range is detected. The magnetic field dependency of the amplitude remains strong also at high pump powers above 2 W where there is no net gain. This again confirms induced absorption by the green pump laser and the interplay between stimulated emission and the induced absorption as discussed previously in this section. The magnetic field reduces the gain in addition to the induced absorption.

As a next step, we investigate the potential for high signal output of the system. The magnetic field sensitivity of the system is improved by both an increased contrast and a high detected signal power (*13*). Because of the highly reflective mirrors, the transmitted signal of the cavity in our configuration is weaker than the reflected signal at the diamond surface (see det2 in Fig. 1A). The reflected signal is caused by birefringence losses turning the p-polarized light without reflection losses under Brewster’s angle to partially reflected s-polarized light. Because of the low absorption sample, these losses become substantial.

The detection of the reflected and transmitted signal is shown in Fig. 4D. The data show that the detected signal for the transmitted amplitude is at ≈1 mW. By detecting the reflection, the signal enhances to ≈8 mW. This enables the detection of stimulated emission signals from an NV ensemble with a milliwatt strength. Thus, by using the cavity laser output, we increase the signal strength substantially compared to the PL detection, which has been used for sensing applications to date. We point out that the cavity signal exits as a beam and can be directed to a distant detector (see Fig. 1A), while the PL requires collection via a microscope objective close to the sample.

### ODMR and sensitivity measurement

So far, we have demonstrated an increased contrast to an applied transverse permanent magnetic field *B_x_* in cavity-assisted readout of the NV centers compared to the PL detection. To predict magnetic field sensitivities of an experimental setup, the technique of ODMR is generally used.

In an ODMR measurement, the frequency of an applied microwave field is scanned over the *m_s_* = 0 → *m_s_* = ±1 transition (Fig. 1B) while the optical emission signal from the NV centers is recorded. The Rabi oscillatory driving field is mixing the spin states and creates a change in PL or rather stimulated emission. The Rabi driving thus takes on the same role as the transverse magnetic field *B_x_* before. In the interaction picture Hamiltonian of constant Rabi driving with a magnetic field amplitude *B*_Ω_ = *B*_1_ cos (ω), the amplitude *B*_1_ plays the equivalent role to the transverse component *B_x_* of the permanent magnet used in the previous measurements (section S9). Thus, we also expect to see an increased contrast and a higher detection signal output in the cavity readout compared to the PL measurement. The magnetic field sensitivity should be improved compared to conventional NV center ODMR via PL.

To apply the microwaves, a loop antenna is brought close to the pumped NV centers in the cavity mode from one side of the diamond as depicted in Fig. 1A. As a first step, we investigate the reachable contrast of the ODMR measurement. Thus, we go to a low seeding power as predicted by our simulation in Fig. 3. We compare the conventionally used technique that detects the PL, i.e., the spontaneous emission of the NV centers to our new technique, which uses the signal enhanced by stimulated emission in the cavity.

**Fig. 5.**
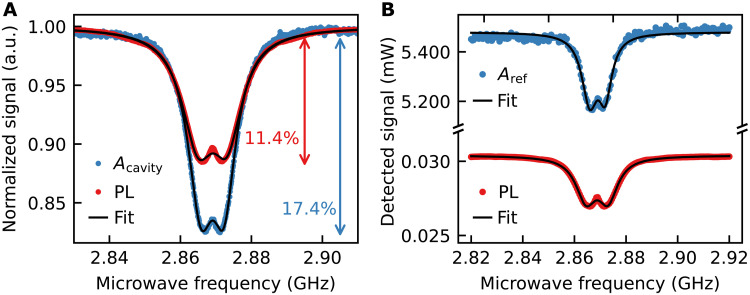
ODMR via stimulated emission. (**A**) Normalized detected power of the transmitted cavity amplitude (*A*_cavity_, det1) and PL signal (det3). The arrows show that the cavity mode has better contrast than the simultaneous PL measurement. (**B**) The detected absolute power of the reflected cavity amplitude (*A*_ref_, det2) is much higher than the PL signal, when optimized for high power. A double Lorentzian is fitted to the data for all cases (black lines).

Figure 5A shows the ODMR measurement of the transmitted (det1) and PL signal (det3). The pump and seeding powers were *P*_532_ = 2.78 W and *P*_710_ = 1.51 mW, respectively. The overall contrast of the ODMR PL signal is 11.4% and 17.4% for our cavity setup. We fitted a double Lorentzian to the data. The parameters for the single Lorentzian with maximal contrast were *C*_PL_ = (8.9 ± 0.0)% and *C*__cavity__ = (13.6 ± 0.1)% with a corresponding ODMR linewidth ∆ν_PL_ = (11.26 ± 0.04) MHz and Δ*v*__cavity__ = (8.92 ± 0.08) MHz for the PL measurement and transmitted cavity signal, respectively. As expected, the contrast of our cavity setup exceeds the PL contrast. In addition, the ODMR linewidth is decreased compared to the PL measurement as well. We explain the reduced contrast in both cases compared to the permanent magnetic field contrast by an inhomogeneous distribution of the magnetic field amplitude over the diamond medium. The achieved record contrast in the previous section is an indication of the achievable ODMR contrast, when the microwave delivery is achieved homogeneously over the cavity volume.

The ODMR measurement allows us to determine and directly compare the sensitivity that is reached by a PL measurement and our cavity-enhanced sensing. The shot noise–limited DC sensitivity of a magnetic field *B* measured via a light signal tI in photons per second in an ODMR measurement from NV centers is $\eta =\partial B/\partial I\sqrt{I}\propto \Delta \nu /(C\sqrt{I})$ (*18*, *39*–*41*). The sensitivity can therefore be improved by narrowing the linewidth Δν of the ODMR or increasing the contrast *C* or detected signal *I*. The output of the cavity readout can be increased with the seeding power, and Fig. 4B indicates that the contrast only reduces slowly, while the signal can be strongly enhanced. For improved sensitivity, we, therefore, go into a regime of higher seeding power above *P*_710_ = 1 W. The measurements are shown in Fig. 5B. We determine the sensitivities from the fit parameters of the ODMR (section S6 and table S1). The resulting shot noise–limited DC magnetic field sensitivity of the PL measurement is $\eta_{\text{PL}}=(276.0\pm 4.7)\;\text{pT}/\sqrt{\text{Hz}}$. The precision is calculated from the SD errors of the fit parameters. For our coherent cavity readout, the DC sensitivity is $\eta_{\text{cavity}}=(29.1\pm 2.5)\;\text{pT}/\sqrt{\text{Hz}}$, that is, an improvement of almost one order of magnitude.

The better sensitivity in stimulated emission–enhanced magnetic field sensing compared to the PL detection is based on a much higher signal through the cavity output *I*_c_ = 5.48 mW compared to the PL detection *I*_PL_ = 30.39 μW and a reduced linewidth (Δν_c_ = 3.11 MHz, Δν_PL_ = 4.60 MHz) in the ODMR. The contrast of the cavity *C*_c_ = 4.20% is reduced in the high seeding power regime compared to the PL *C*_PL_ = 8.95%. This is due to a reduced contribution of the NV center stimulated emission to the cavity signal compared to the signal contribution of the seeding laser, as previously calculated (see Fig. 3). The contrast is only slowly decreasing with increasing seeding power (see Fig. 4B) and leads to an improved sensitivity compared to the PL. In future work, increased material thickness or NV density could help uphold the contrast advantage in higher seeding power regime.

## DISCUSSION

We have shown light amplification by stimulated emission from NV centers of ≈64% in a macroscopic laser cavity with an optimal pump power of 1 to 2 W at a red seeding power of <0.2 W. While previously stimulated emission from NV centers was detected via a lock-in amplifier (*22*) or single-photon detectors (*27*), we achieved stimulated emission signals with an absolute power in the milliwatt range. We show that induced absorption by the green pump laser is an additional loss at the red lasing wavelength of 710 nm that leads to a decrease of the amplification at high pump powers >2 W. This additional loss path is identified as a purely material-related absorption loss induced by the green laser because the linewidth and finesse are reduced, i.e., loss increased while the free spectral range is constant. Possible candidates could be ionization to the conduction band and tunneling of the electron to other defects (e.g., nitrogen or nitrogen complexes) leading to a reduction in quantum efficiency and the creation of different charge states of defects that absorb at this wavelength.

Furthermore, we detect magnetic field dependency of the amplification by stimulated emission of the NV centers. The resulting contrast in the amplitude of the cavity resonance is detected to be 26 to 33% at an output power between 0.1 and 8 mW, depending on the detection of the transmitted and reflected signal of the cavity. The achieved ensemble contrast is a new record for NV centers and is higher than what can be achieved with spontaneous emission. Thus, we demonstrate experimentally the advantages of coherent cavity readout for sensing and the principle of LTM for the first time. This opens the door for a variety of sensing techniques and applications, precision improvements in sensors, and the exploration of coherent readout of quantum systems for sensing, quantum bits, and quantum technology more generally. The detection of ODMR with coherent laser output shows a magnetic field sensitivity of $(29.1\pm 2.5)\;\text{pT}/\sqrt{\text{Hz}}$, which is an improvement of almost one order of magnitude compared to the conventional PL readout.

For further improvement and the development of a highly sensitive magnetic field sensor, a sensitivity measurement can be done via locking the cavity. Cavity locking could be achieved by the Pound-Drever-Hall technique, modulating the seeding laser with an electro-optic modulator and detecting the transmitted or reflected signal. The ODMR measurement can then be combined with a continuous wave signal detection. An increase in the signal output is expected by impedance matching the mirrors. In addition, via lock-in detection of the NV emission, the red seeding laser could be filtered out, and amplification and contrast measurements indicate that a contrast of almost unity might be reached. In addition, the development of a microwave antenna, e.g., a loop antenna on both sides of the diamond or a microwave resonator, could lead to a more homogeneous microwave field and an increased ODMR contrast.

## MATERIALS AND METHODS

### Diamond material

The sample used for our studies is a (100) oriented, both sides polished, commercial high-pressure, high-temperature (HPHT) diamond from Element Six with an edge length of 3 mm and a thickness of *l* = 295 μm. It is treated with low-pressure, high-temperature (LPHT) annealing at 1800^°^C in an inert gas atmosphere to reduce the initial absorption before electron irradiation with a fluence of 1 × 10^18^ cm^−1^ and an electron energy of 2 MeV. After irradiation, the sample was again annealed at 1000^∘^C for 2 hours to create NV centers.

### Experimental setup

For all measurements, we used the setup in Fig. 1A. We used lenses to prepare the beam size for optimal mode matching of the seeding laser and matching the pump beam to be slightly larger than the cavity mode. To efficiently couple into the cavity mode and pump the NV centers, we horizontally polarized both beams as the diamond was placed in Brewster’s angle.

We have taken several actions to stabilize the complete system. To measure the finesse, the cavity needs to be scanned, which prevents active locking. However, highly stable wavelength locking of the seed laser to an external cavity to 710.0000 nm was implemented, as well as a closed-loop scanning piezo system working in scanning mode providing the (relative) piezo position as feedback, experiment construction with high-mass elements to reduce vibrations and high piezo scanning frequency (111 Hz) to remove the influence of acoustic noise on the fast mirror transition through cavity resonance and free spectral range. The cavity length was modulated by applying a sawtooth signal with an amplitude of 3 μm. Several fundamental modes of the cavity were sampled with an oscilloscope. The cavity resonances were detected in the linear regime of the piezo movement. We detected the power of the transmitted (det1) and reflected (det2) cavity signal through variable gain photoreceivers. The photoreceivers were power-calibrated. Immediate analysis by integrated oscilloscope functions enabled a fast readout of the measurement quantities, i.e., the cavity resonance amplitude, FWHM, and FSR.

A photodiode power sensor directly measured the power of the PL (det3) spectrally filtered by a 600-nm long-pass filter to reduce the NV^0^ contribution. The use of the microwave antenna for the ODMR measurement leads to a decrease in the PL signal because of partially blocking the PL light. To have comparable PL measurements, we used a saturation measurement of the PL without microwave antenna where the PL collection was maximized. We used this measurement as a calibration of the PL signal (fig. S5).

## Supplementary Materials

### This PDF file includes:

Sections S1 to S9 Figs. S1 to S8 Table S1 References
