# Supplementary material for: Magnetic-field-dependent stimulated emission from nitrogen-vacancy centers in diamond
Source: Sci Adv. 2022 Jun 3;8(22):eabn7192. doi: 10.1126/sciadv.abn7192 (PMC9166290; doi:10.1126/sciadv.abn7192)
Supplement: Supplementary file 1 — Sections S1 to S9 Figs. S1 to S8 Table S1 References [file sciadv.abn7192_sm.pdf]

Science Advances
AAAS

Supplementary Materials for

Magnetic-field-dependent stimulated emission from nitrogen-vacancy centers in diamond

Felix A. Hahl *et al.*

Corresponding author: Felix A. Hahl, felix.hahl@iaf.fraunhofer.de; Jan Jeske, jan.jeske@iaf.fraunhofer.de

Sci. Adv. **8**, eabn7192 (2022)
DOI: 10.1126/sciadv.abn7192

This PDF file includes:

Sections S1 to S9
Figs. S1 to S8
Table S1
References

S1. Investigation of the cavity parameters

We investigate the origin of the amplification by the detection of the cavity amplitude, FSR and FWHM during a scan of the pump power in Fig. S1. The measurement proves that the increase of the cavity finesse (orange trace) comes from gain caused by stimulated emission reducing the total loss of the cavity (blue trace). The gain shown in the main article is calculated from the finesse with formula $\mu_g = \pi/l(\mathcal{F}_0^{-1} - \mathcal{F}_g^{-1})$. The value of the finesse at zero pump power is $\mathcal{F}_0 = 847$ and the maximal value when pumping is $\mathcal{F}_{max} = 1011$. The FSR is constant as expected. This shows that no thermal effect, like thermal expansion of the diamond is influencing the FSR of the cavity. Stimulated emission, should also lead to an increasing

number of photons n in the cavity, not just a narrowing of the peak width. Thus, an increase in the area $S \propto n$ is expected, as well. The Lorentzian amplitude A is given by $A = 2S/(\pi\mu)$ with the area S under the curve and the total gain/loss $\mu = \mu_0 L - \mu_g l$. Thus, the area is calculated from the measured amplitude and FWHM and shown in Fig. S1. The increase in the area proves the increase in the number of photons in the cavity as expected from stimulated emission.

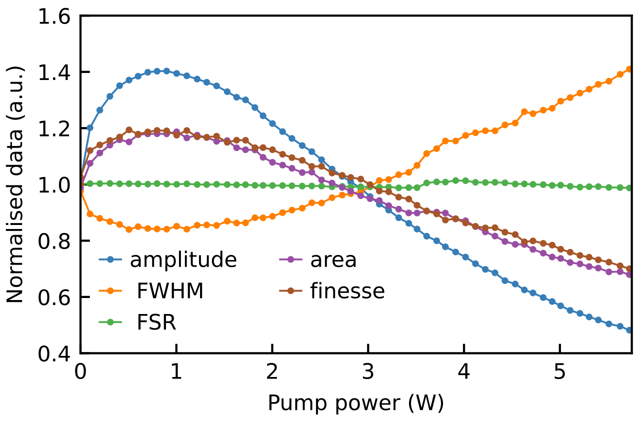

Fig. S1. Light amplification and cavity parameters dependent on pump power. Detection of the amplitude, FSR and FWHM of the cavity modes over the pumping power. Area and finesse are calculated by the other measured quantities (see text). The data is normalised to the values at zero pump power. The data show the mean value of 100 measurements. The seeding power is $P_{710} = (123.5 \pm 0.8)\,\mathrm{mW}$.

S2. Magnetic field of the permanent magnet

We measured the magnetic field with a Hall sensor at varying distance from the magnet. The magnetic field is applied in the (100) crystal direction. The diamond contains NV-centres in all four possible NV-directions. In this configuration the projections of the applied magnetic field onto and perpendicular to the NV-axis is symmetric and of the same magnitude for all NV-orientations. The angle between the NV-axis is $\alpha = 109.5°$ (*21*). The effective transverse magnetic field that the electron spin is interacting with is then calculated by $B_x = B\sin(\alpha/2)$,

where B is the magnitude of the measured magnetic field. We assume a constant magnetic field within the mode of the diamond of diameter $2\omega_0 \approx 110\,\mu\mathrm{m}$. The resulting effective magnetic field is shown in Fig. S2. The excited NV-centres in the diamond mode in the experiment are located at a distance of $(2 \pm 1)\,\mathrm{mm}$ from the magnet. The effective transverse magnetic field is $B_x = (146 \pm 25)\,\mathrm{mT}$.

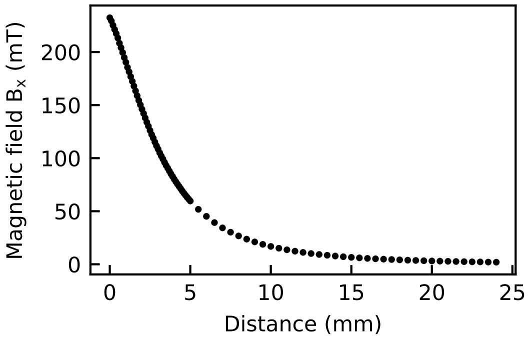

Fig. S2. Transverse magnetic field strength of the permanent magnet. The effective transverse magnetic field B_x (perpendicular to the NV-axis) as a function of the distance to the permanent magnet.

S3. NV PL contrast by a constant transverse magnetic field B_x

Stimulated emission in a cavity can significantly enhance the contrast, theoretically up to $100\,\%$ when working above a laser threshold (*22*). The strong contrast observed in the main article in stimulated emission is higher than the maximal contrast possible in spontaneous emission according to the current model of the NV centre, as we show below. We calculate theoretically the change of spontaneous emission, i.e. the photoluminescence contrast of the NV-centres as a function of a constant, external magnetic field perpendicular to the NV direction. Without loss of generality, the perpendicular magnetic field can be chosen to the match the direction of transverse magnetic field component B_x and leads to mixing spin states.

We assume the 7-level model of Fig. S3A for the calculation. Following established litera-

ture (*21*), we assume the radiative decay rate $\Gamma = 83.3\,\mathrm{MHz}$, the intersystem crossing rate from the ± 1 states to the singlet state $L_1 = 1/(2 * 12\,\mathrm{ns} + 0.9\,\mathrm{ns})$ as half of the radiative decay rate, no transition from the $m_s = 0$ state to the singlet state, the rate $L_3 = 2.2\,\mathrm{MHz}$ and $L_2 = L_3/2$, such that the lifetime of the singlet ground state becomes $1/(L_2 + L_3) = 308\,\mathrm{ns}$. The singlet state is just represented by one state. However, it has an additional short-lived excited state with a lifetime $\approx 0.9\,\mathrm{ns}$ (*21*), which we integrated into the rate L_1 to simplify the calculation.

Assuming the weak excitation limit, we consider the effect of magnetic field on the ground-state spin triplet only, and ignore dynamics and coherence in the excited-state triplet. The ground state triplet with spin operators $\vec{S}$ in an external magnetic field $\vec{B}$ is represented by the Hamiltonian

$$H^{PL} = D_g S_z^2 + \gamma_e \vec{B}\vec{S} = \begin{pmatrix} \gamma_e B_z + D_g & \gamma_e B_c^* & 0 \\ \gamma_e B_c & 0 & \gamma_e B_c^* \\ 0 & \gamma_e B_c & -\gamma_e B_z + D_g \end{pmatrix} \tag{S.1}$$

where $B_c = (B_x + iB_y)/\sqrt{2}$ represents the magnetic field perpendicular to the NV direction, D_g the zero-field splitting of the ground state and γ_e the electron gyromagnetic ratio. The X-axis can be chosen in the direction of the transverse magnetic field, making $B_c = B_x/\sqrt{2}$ a real number. We then construct a superoperator matrix describing the quantum evolution of the 3x3 density matrix for the ground-state triplet according to the von-Neumann equation. We then extend the 9x9 superoperator describing the coherent evolution to a 13x13 matrix describing the populations of the other 4 states. We do not consider any further coherences. The transition rates are then integrated via a Lindblad equation with a Lindblad-operator $L_j = \sigma^-$ on the corresponding two states. We then find the steady state solution of the system analytically by calculating the Nullspace of the superoperator matrix using Mathematica. The emission of the NV centre is then given by the sum of the populations in the triplet excited state multiplied with the radiative decay rate Γ.

We find that the NV emission is reduced by a transverse magnetic field as shown in Fig. S3B.

This relative reduction is true for all excitation rates $\Lambda \ll \Gamma$ in the weak-excitation regime, for which this solution is valid. Since we are pumping a cavity mode with $111\,\mu\mathrm{m}$ diameter even a high pump power of $3\,\mathrm{W}$ is still in the weak-excitation regime $\Lambda < 0.1\Gamma$ and far from saturation, see Fig. S5. The magnetic field along the NV direction $B_z = 0$ in Fig. S3B. Minimizing the relative emission for arbitrary B_z and B_c we find that the minimum is 0.78, i.e. the strongest reduction is 22%.

This means that the maximal contrast of spontaneous emission of an NV ensemble, caused by the introduction of an external magnetic field is $22\,\%$. The literature parameters of the model (*21*) were developed from mostly measurements of ensembles of NV centres, so the result applies to ensembles and single NV centres can potentially achieve better contrast. Specifically, single NV-centres in a confocal setup can be pumped into a high-excitation limit, which can enhance contrast to $>40\,\%$ when a permanent magnetic field mixes the ground- and excited-state spin populations (*42*). However, for pumping larger volumes of NV ensembles even high powers typically stay in the weak-excitation limit, as is the case for all our measurements. The strong contrast in ensembles measured through stimulated emission in the main article is stronger than a contrast could be with spontaneous emission according to the current model for NV centre ensembles.

S4. Amplification and absorption study of different samples

In Fig. S4A the relative amplification, absorption at the seeding wavelength of $710\,\mathrm{nm}$ and NV^- concentration are shown for four different samples that were investigated. The data shows that both, low absorption and high NV^- concentration, i. e. high gain, is needed for a strong stimulated emission signal. For decreasing absorption the amplification is increasing, as the loss inside the cavity is decreasing. At the same time the amplification is also dependent on the gain which is proportional to the number of pumped NV-centres (*37*). The highest amplification

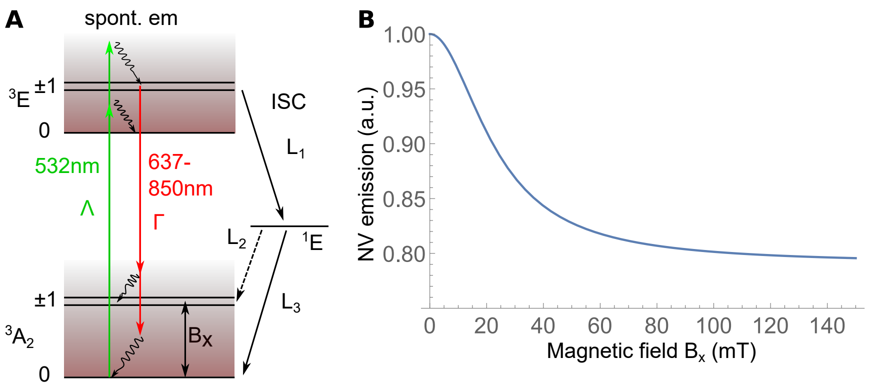

Fig. S3. PL emission quenching by a transverse magnetic field. (**A**) Energy levels and transitions of the NV centre. (**B**) Calculated PL emission of the NV-centre dependent on the transverse magnetic field strength. The PL reduction saturates at $22\,\%$.

is measured for the lowest absorption and highest NV^- concentration. For equal NV^- concentration a stronger amplification is detected when the absorption is lower. A very low NV^- concentration ($50\,\mathrm{ppb}$) leads to no significant gain/amplification. Fig. S4B shows the calculated population of the NV-centre ground state phonon levels as a function of the temperature. The population of the k-th phonon sideband p_k is normalised to the zero phonon level p_0 and given by the Boltzmann distribution $p_k = \exp\left(\Delta E_k/(k_B T)\right)$, where k_B is Boltzmann's constant, T temperature and ΔE_k, the energy difference of the k-th phonon level to the zero phonon level. The energy differences are taken from (*43*). The population of the third phonon level is below $1\,\%$, consequently the absorption of the red seeding laser at this transition is negligible.

S5. Saturation measurement - PL calibration

We collected the PL with an objective laterally to the pumped NV centres (main article, Fig. 1B) and measured the power with a photo-diode power sensor (det3). The PL was maximised for this measurement. We fitted the function $\mathrm{P_{PL}} = \mathrm{P_{inf}}\mathrm{P_{pump}}/(\mathrm{P_{pump}} + \mathrm{P_{sat}})$ to the data with the fitting parameters $\mathrm{P_{inf}}, \mathrm{P_{sat}}$. The fit gave $\mathrm{P_{inf}} = 587.68\,\mu\mathrm{W}$ which is the PL power at infinite pump power and $\mathrm{P_{sat}} = 70.61\,\mathrm{W}$ which is the pump power at saturation. In the ODMR measurement

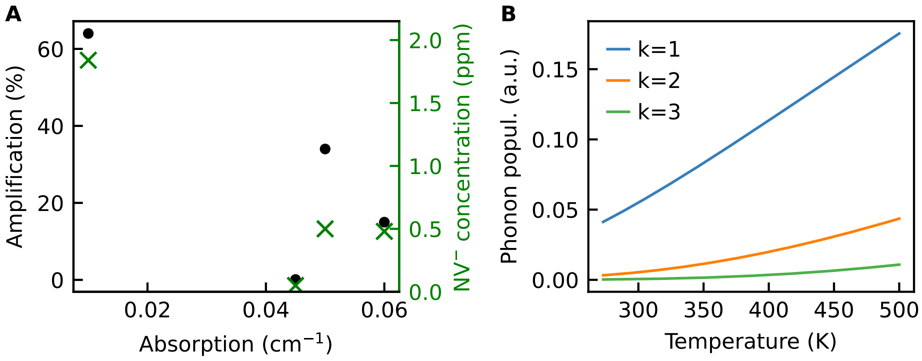

Fig. S4. Influence of initial absorption of the diamond and NV centres. (**A**) Data pairs of the maximal achieved relative amplification in the cavity (black dots) and NV^- concentration (green crosses) for four different investigated samples over the absorption. The decadic absorption coefficient is measured at $710\,\mathrm{nm}$ via UV-Vis measurement of the whole sample taking into account the thickness and reflection. The NV^- concentration was calculated from a calibrated PL measurement in a confocal setup. (**B**) Simulated population of the $k = 1st, 2nd, 3rd$ phonon side-band over the temperature.

the antenna partially blocked the PL signal. For a fair comparison, we therefore used the fit of Fig. S5 and the green pump power to determine the corresponding absolute PL signal for Fig. 5 of the main article.

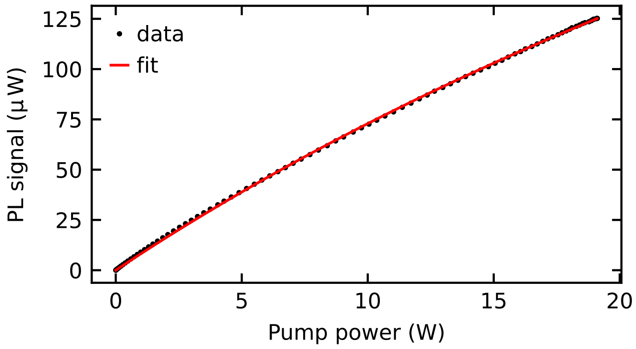

Fig. S5. Saturation measurement of the PL emission. The PL of the NV centres is detected while the laser power is increased. The amplification measurements (up to $4\,\mathrm{W}$) are conducted in the linear regime.

S6. ODMR magnetic field sensitivity

In measurements of the magnetic field B in the standard quantum limit the measurement error, i.e. the standard deviation $\sigma_B \propto 1/\sqrt{T}$, reduces with longer measurement time T. Therefore, the definition of the DC magnetic field sensitivity captures precision independent of the measurement time and is defined as $\eta_{DC} = \sigma_B\sqrt{T}$. Assuming photon shot-noise limited sensitivity, it can be calculated directly from the intensity I in counts per second as

$$\eta_{DC} = \frac{\partial B}{\partial I}\sqrt{I} = \frac{h}{g_e\mu_B}\left|\frac{\partial I}{\partial f}\right|^{-1}\sqrt{I}. \tag{S.2}$$

In the second step the optically detected magnetic resonance (ODMR) measurement is considered where the intensity is recorded as a function of the microwave frequency f. The fundamental constants h, g_e, μ_B are Planck's constant, the electron g-factor and Bohr magneton, respectively. This yields the sensitivity at each point of the ODMR curve as a function of the intensity gradient $\partial I/\partial f$ and the intensity I at that point. The minimum of this sensitivity can be calculated for Lorentzian line shapes as (*39, 40*)

$$\eta_{DC} \approx \frac{4}{3\sqrt{3}}\frac{h}{g_e\mu_B}\frac{\Delta\nu}{C\sqrt{I}}, \tag{S.3}$$

where $\Delta\nu, C, I$ are the linewidth (FWHM), contrast and detected signal strength of the measurement, respectively. For the sensitivity calculations in the main article the formula (S.3) is used. The intensity I is calculated from the detected power P_0 as $I = P_0\lambda/(ch)$, where c is the speed of light and $\lambda = 710\,\mathrm{nm}$ the wavelength of the detected signal. We obtain the detected power from power calibrated detectors (Sec. Methods in main article). The pump power was $3.75\,\mathrm{W}$, the seeding power was $1.32\,\mathrm{W}$. This regime was not optimal for the stimulated emission contrast since the strong seeding laser is not filtered from the signal but achieved much higher output powers and thus improved sensitivity due to stronger stimulated emission from

the increased seeding. A double Lorentzian

$$P(\nu) = P_0 \left(1 - \frac{C_1 \Delta\nu_1^2}{(\nu - \nu_{c,1})^2 + \Delta\nu_1^2} - \frac{C_2 \Delta\nu_2^2}{(\nu - \nu_{c,2})^2 + \Delta\nu_2^2}\right), \tag{S.4}$$

is fitted to the data in the main article (Fig. 5). Here $\nu, P_0, C, \Delta\nu, \nu_c$ are the microwave frequency, baseline, contrast, HWHM and centre frequency, respectively. The index represents the number of the single Lorentzian. The data is shown in Fig. S6. The plots also show the contributions of the single Lorentzian fits. The related fit parameters are listed in Table S1. The parameters for the calculation of the setup sensitivities were taken from column C, index 2 for the PL measurement and from column D, index 2 for the cavity setup.

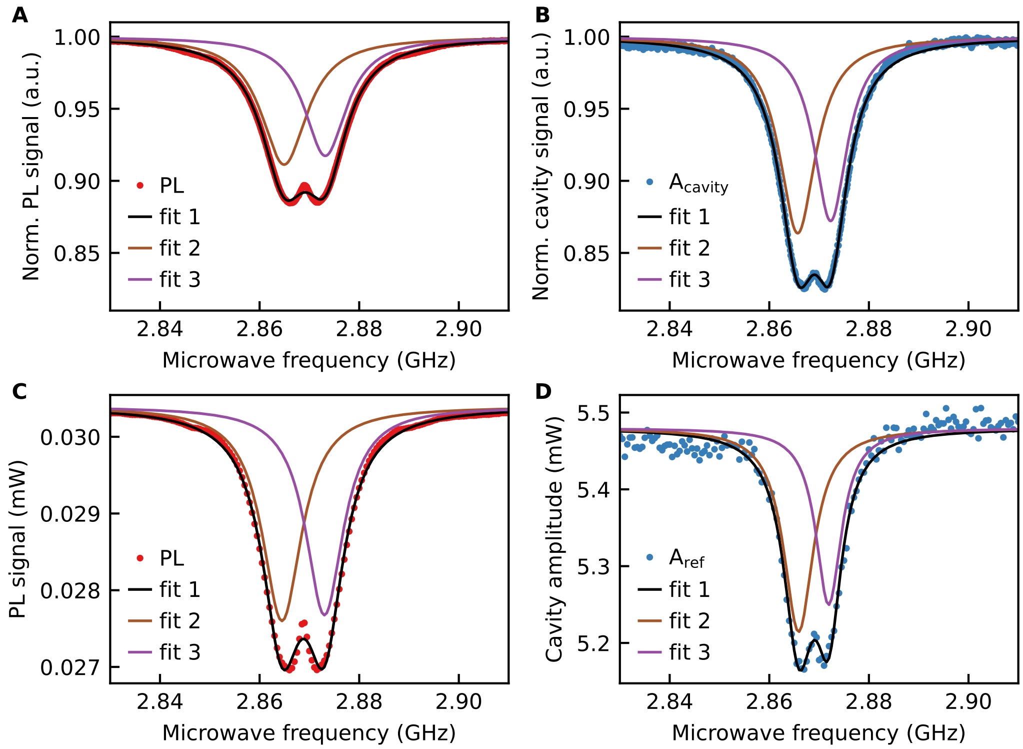

Fig. S6. Lorentzian fits to ODMR measurement. ODMR measurement optimised for high contrast ((**A**), (**B**)) and optimised for sensitivity ((**C**), (**D**)). For every dataset a double Lorentzian (fit1) and the contribution of the single Lorentzian fits (fit2, fit3) is fitted to the data.

Table S1. ODMR fit parameters. Fit parameters obtained from the double Lorentzian fits in Fig. S6. The header line indicates the related plot.

Fit Fig. S6	A	B	C	D
P_0	$1\,(\mathrm{a\,u})$	$1\,(\mathrm{a\,u})$	$30.39\,\mu\mathrm{W}$	$5.48\,\mathrm{mW}$
$C_1(\%)$	(8.87 ± 0.03)	(13.64 ± 0.09)	(9.20 ± 0.08)	(4.84 ± 0.18)
$C_2(\%)$	(8.23 ± 0.03)	(12.79 ± 0.10)	(8.95 ± 0.09)	(4.20 ± 0.20)
$\Delta\nu_1(\mathrm{MHz})$	(5.63 ± 0.02)	(4.46 ± 0.04)	(4.81 ± 0.07)	(3.64 ± 0.21)
$\Delta\nu_2(\mathrm{MHz})$	(5.16 ± 0.02)	(4.07 ± 0.04)	(4.60 ± 0.07)	(3.11 ± 0.23)
$\nu_{c,1}(\mathrm{GHz})$	2.865	2.866	2.864	2.867
$\nu_{c,2}(\mathrm{GHz})$	2.873	2.872	2.873	2.872

S7. Ionisation and induced absorption

Fig. S7A shows the relative concentrations of negatively and neutrally charged NV centres and thus the photoionisation over the pump power. A least squares fit method determines the relative contributions of the charge states to the measured spectrum. The basis are reference NV^- and NV^0 spectra measured in the same setup. The method fits the reference spectra to the measured original PL spectrum taken from the side (det3, Fig. 1A in the main article).

Fig. S7B shows the timetrace of the maximal cavity amplitude on resonance when the green pump laser is applied and turned off again. The time scale of the immediate amplification from stimulated emission is below the temporal resolution $< 1\,\mathrm{ms}$, limited due to the unlocked cavity setup and read-out of the cavity amplitude. The amplitude also immediately decays when the pump laser is turned off, as expected for stimulated emission. However, when the pump is turned off the decay of the cavity amplitude goes below the initial value before pumping. This means less red light is transmitted through the cavity after the green laser illumination than before. In other word an additional absorption at the seed wavelength of $710\,\mathrm{nm}$ is 'induced' by the green pump laser. After the green laser is turned off and the signal has immediately dropped to a value lower than the initial value, the signal starts to rise again slowly back to the initial

value before green pumping. The decay time of the "induced absorption" is in the order of several seconds $\tau_{decay} = 5.7\,\mathrm{s}$. The temporal behaviour indicates that we are not in the regime of excited state absorption due to the NV^{-} excited state lifetime of $12\,\mathrm{ns}$ (*21*).

However, ionisation occurs with increasing pump power as shown in Fig. S7A. A certain reduction of the NV- population can be seen although it is still above $80\,\%$ of the initial value for pump powers below 5W. While this could explain a small reduction in gain from NV- centres, it can not be the reason for the induced absorption of the red laser since the created NV^{0} centres do not absorb light at $710\,\mathrm{nm}$. We think the ionisation plays a different role in this novel effect.

We suggest the following explanation: The photoionisation of the NV centres leads the creation of certain charge states of other defects or in other words leads to a population of long lived intra band gap states. These states absorb light at the seeding wavelength of $710\,\mathrm{nm}$. A possible candidate could be the H2/H3 centre in diamond as the H2 centre absorbs at this wavelength (*44*).

The resulting absorption induced by the pump laser is higher than the initial absorption before the NV centres were pumped.

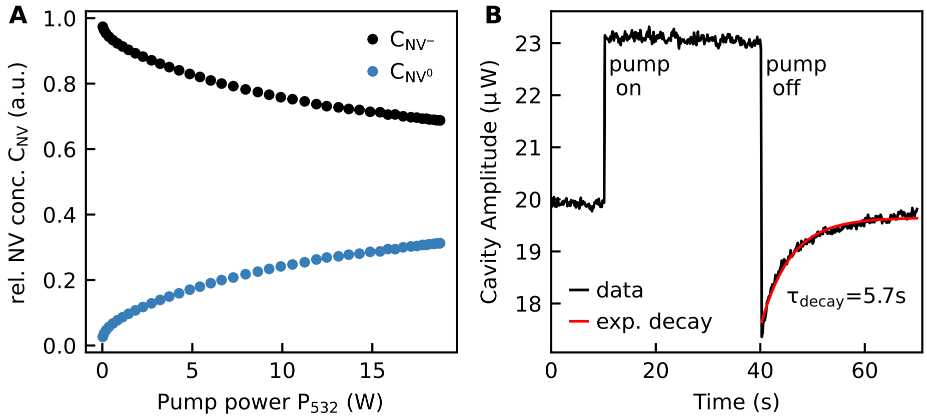

Fig. S7. Effect of ionisation and induced absorption on light amplification. (**A**) Relative NV concentration over pump power P_{532} of the sample used in the main article. The point of minimal pump power corresponds to $C_{NV^-} = 1.8\,\mathrm{ppm}$ and $C_{NV^0} = 0.07\,\mathrm{ppm}$. (**B**) Investigation of the amplification and decay time scales. Detected is the cavity amplitude over time. The red seeding is kept constant at $P_{seed} = 610\,\mathrm{mW}$. After $10\,\mathrm{s}$ the pump laser is applied at a pump power of $P_{532} = 260\,\mathrm{mW}$ and turned off after another $30\,\mathrm{s}$. An exponential function $y = a * \exp(-t/\tau_{decay}) + b$ is fitted to the slow decay when the pump laser is turned off.

S8. Analytic solution of an externally seeded NV cavity

For the seeded NV lasing cavity we assume the 7-level model presented in Fig. S8A with a constant excitation rate Λ and coupling constant between the NV centres and the cavity G for both spin states. We set up the equations of motion describing the populations of the 7-level system as a superoperator matrix and assume the transition rates by the standard NV model as in (*21, 22*). The matrix and transition rates are given at the last page of the Supplementary Material. The simulation of the seeded cavity is based on a realistic cavity with a finesse of $\mathcal{F} = 800$ leading to a loss rate of $\kappa \approx 80\,\mathrm{MHz}$ corresponding to the cavity used in the main article. The coupling constant G between the NV centre and cavity is chosen to be $G = 6\,\mathrm{GHz}$ which corresponds to the theoretically expected coupling $G = 3\Gamma\omega_{seed}\lambda_m^3/(4\pi^2\Delta\omega_3 V_c)$ (*37*) at the 3-phonon transition of the NV centre with a linewidth of $\Delta\omega_3 = 15\,\mathrm{THz}$ (*34*), spontaneous emission rate $\Gamma = 83\,\mathrm{MHz}$ angular frequency $\omega_{seed} = 2.65\,\mathrm{PHz}$ and medium wavelength $\lambda_m =$

λ_{seed}/n_d of the seed laser with $\lambda_{seed} = 710\,\mathrm{nm}$ and a cavity mode volume of $V_c = 0.14\,\mathrm{mm}^3$.
The cavity rate equation (S.5)

$$\dot{n} = G(\rho_{22} + \rho_{55} - \rho_{33} - \rho_{66})n - \kappa n + \alpha \tag{S.5}$$

describes the seeded cavity with seeding rate α and number of cavity photons per NV centre n.
In order to find the solution of the system we first calculate the steady state solution, i.e. the nullspace of the superoperator matrix, treating n as a steady-state parameter. We solve for two cases: no applied magnetic field $B_x = 0$ and large magnetic field $\gamma_e B_x \gg D_g$, where the spin states are fully mixed by the transverse magnetic field. We normalise the resulting solutions for the density matrix elements ρ_{ij} via the sum of the diagonal elements ρ_{ii} and insert these into the cavity rate equation (S.5) for the steady state

$$0 = G(\rho_{22} + \rho_{55} - \rho_{33} - \rho_{66}) - \kappa + \frac{\alpha}{n} \tag{S.6}$$

to find the non-zero solution for $n \neq 0$.
We then simulate the behaviour of the system with an applied transverse magnetic field B_x via the stimulated emission rate of the cavity by $\Gamma_{stim} \propto \rho_e G n$, where the excited state population is $\rho_e = \rho_{22} + \rho_{55} - \rho_{33} - \rho_{66}$.
The simulation in the main article in Fig. 3 calculates the contrast by stimulated emission via the solution at zero magnetic field $B_x = 0$ and strong magnetic field, i.e. $D_g \ll \gamma_e B_x/\sqrt{2}$. We assume a constant excitation rate $\Lambda = 1\,\mathrm{MHz}$ in the low excitation regime leading to a cavity below threshold as in the experiment. The simulation in Fig. S8B shows an increasing contrast by stimulated emission when the seeding is low, i.e. the contribution of the stimulated emission to the overall cavity signal is large. This is what is also measured experimentally in the main article in Fig. 4C and Fig. 5A. In this regime the contrast by stimulated emission exceeds the maximal achievable contrast via PL of 22%.

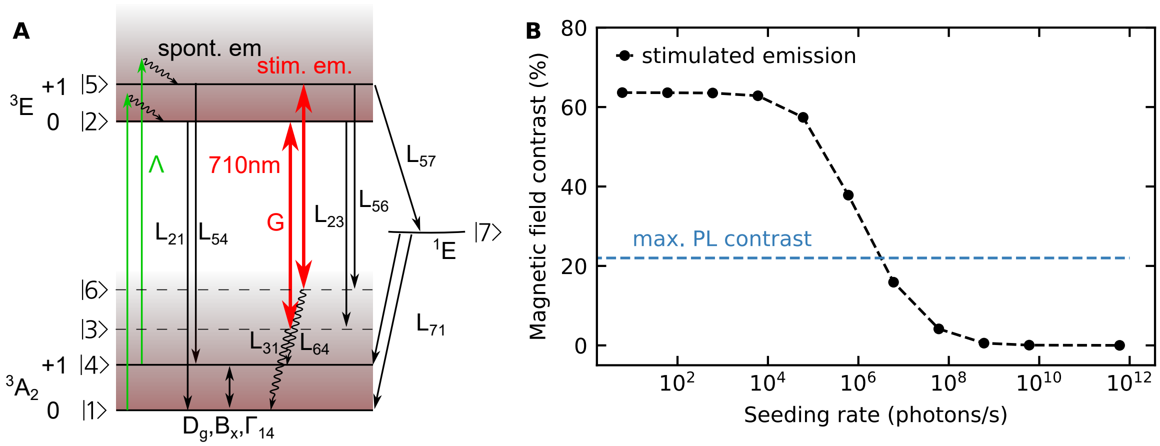

Fig. S8. Modelling magnetic field response of seeded laser cavity below threshold. (**A**) Schematic of the NV 7-level-model describing the lasing transitions between the $m_s = 1$ and $m_s = 0$. The seven states are numbered by $|X\rangle$ and the transition rates between the states by L_{XX}. Stimulated emission is generated by the coupling between the NV and the cavity G. The ground state is split by the zero field splitting constant D_{g}. The dephasing rate between the ground spin sates is given by Γ_{14}. The applied transverse magnetic field B_x leads to spin-mixing similar to the PL case in Fig. S3A. (**B**) Magnetic field contrast over the cavity seeding rate α calculated by the method presented in Sec. S8. The maximal PL contrast is calculated by the method in Sec. S3.

S9. NV PL contrast by MW driving and similarity to transverse magnetic field B_x

In the main article we used two experiments/methods to mix the spin states $m_s = 0, \pm 1$ of the NV centre ensemble in the bulk diamond sample. The first (method 1) uses a constant transverse magnetic field $B_c = B_x/\sqrt{2}$ applied by a permanent magnet (section 'Magnetic field dependency'). The theoretical calculation (Ch.S3, supplementary material) calculates the PL quenching due to spin mixing which results from the off-diagonal $B_c = B_x/\sqrt{2}$ components which equals the transverse component in the experiment.

The second method (method 2) uses the ODMR technique (section 'ODMR and sensitivity measurements' in the main article), where the Rabi driving by an applied oscillating magnetic field $B_\Omega(t) = B_1 \cos(\omega t)$ with constant amplitude B_1 and frequency ω is mixing the spin states

and creates the decrease in PL or rather stimulated emission.

In the interaction picture of constant Rabi-driving with a magnetic field amplitude B_1, the Hamiltonian (*45*)

$$H^{MW} = D_g S_z^2 - \vec{\mu}\vec{B_\Omega}(t) \overset{RWA}{=} \begin{pmatrix} \gamma_e B_z + D_g - \omega & -\frac{\gamma_e B_1}{\sqrt{2}} & 0 \\ -\frac{\gamma_e B_1}{\sqrt{2}} & 0 & -\frac{\gamma_e B_1}{\sqrt{2}} \\ 0 & -\frac{\gamma_e B_1}{\sqrt{2}} & -\gamma_e B_z + D_g - \omega \end{pmatrix} \tag{S.7}$$

in the rotating wave approximation (RWA) describes the system.

We can now compare the Hamiltonian for a constant transverse magnetic field above (Ch.S3, supplementary material) with the Hamiltonian for a constant microwave (MW) drive H^{MW}. For both experimental methods in the main article the component $B_z = 0$, as the applied field is transverse (method 1) and we don't apply a bias field (method 2).

We now distinguish two cases: 1) The case without spin mixing, i.e. a zero transverse field $B_x = 0$ (method 1) corresponding to a detuned microwave drive $\omega \ll D_g$ or $\omega \gg D_g$ (method 2) and 2) the case with spin mixing, i.e. a strong transverse magnetic $\gamma_e B_x \gg D_g$ (method 1) corresponding to a resonant drive $\omega = D_g$ (method 2). For both cases the Hamiltonians H^{PL}, H^{MW} are mathematically equivalent and the physics of the NV centre is the same. Of course the microwave field B_1 mixes the spin states at much lower field values than a transverse magnetic field, since the transverse field B_x has to overcome the zero-field splitting D_g. However this shows that in the extreme cases the transverse magnetic field component B_x of the permanent magnet plays the equivalent role to the constant microwave amplitude B_1 for mixing the spin states.

In the experiment a transverse magnetic field is the easiest way to test maximal spin mixing due to better homogeneity. Once an improved contrast is measured that way (see main article) we succeeded to see this improved contrast in ODMR as well, which is lower to the permanent magnet as it has the additional challenge of creating a good homogeneity but is the necessary way for good sensitivity.

$$\begin{pmatrix} \dot\rho_{14} \\ \dot\rho_{41} \\ \dot\rho_{11} \\ \dot\rho_{22} \\ \dot\rho_{33} \\ \dot\rho_{44} \\ \dot\rho_{55} \\ \dot\rho_{66} \\ \dot\rho_{77} \end{pmatrix} = \begin{pmatrix} -\Gamma_1-\Lambda & 0 & iB_x & 0 & 0 & -iB_x & 0 & 0 & 0 \\ 0 & -\Gamma_1^*-\Lambda & -iB_x & 0 & 0 & iB_x & 0 & 0 & 0 \\ iB_x & -iB_x & -\Lambda & L_{21} & L_{31} & 0 & 0 & 0 & L_{71} \\ 0 & 0 & \Lambda & a & Gn & 0 & 0 & 0 & 0 \\ 0 & 0 & 0 & L_{23}+Gn & -L_{31}-Gn & 0 & 0 & 0 & 0 \\ -iB_x & iB_x & 0 & 0 & 0 & -\Lambda & L_{54} & L_{64} & L_{74} \\ 0 & 0 & 0 & 0 & 0 & \Lambda & b & Gn & 0 \\ 0 & 0 & 0 & 0 & 0 & 0 & L_{56}+Gn & -L_{64}-Gn & 0 \\ 0 & 0 & 0 & 0 & 0 & 0 & L_{57} & 0 & -L_{71}-L_{74} \end{pmatrix} \begin{pmatrix} \rho_{14} \\ \rho_{41} \\ \rho_{11} \\ \rho_{22} \\ \rho_{33} \\ \rho_{44} \\ \rho_{55} \\ \rho_{66} \\ \rho_{77} \end{pmatrix}$$

$\Gamma_1 = \Gamma_{14} - iD_g$

$a = -L_{21} - L_{23} - Gn$

$b = -L_{54} - L_{56} - L_{57} - Gn$

$L_{21} = L_{54} = 68.2\,\mathrm{MHz}, L_{31} = L_{64} = 1\,\mathrm{THz}, L_{23} = L_{56} = 18\,\mathrm{MHz}, L_{71} = 2.16\,\mathrm{MHz}, L_{74} = 1.08\,\mathrm{MHz}, L_{57} = 40.2\,\mathrm{MHz}, \Gamma_{14} = 0.167\,\mathrm{MHz}, D_g = 2\pi \cdot 2.87\,\mathrm{GHz}$

REFERENCES AND NOTES

1. E. Boto, N. Holmes, J. Leggett, G. Roberts, V. Shah, S. S. Meyer, L. D. Muñoz, K. J. Mullinger, T. M. Tierney, S. Bestmann, G. R. Barnes, R. Bowtell, M. J. Brookes, Moving magnetoencephalography towards real-world applications with a wearable system. *Nature* **555**, 657–661 (2018).

2. R. Zhang, W. Xiao, Y. Ding, Y. Feng, X. Peng, L. Shen, C. Sun, T. Wu, Y. Wu, Y. Yang, Z. Zheng, X. Zhang, J. Chen, H. Guo, Recording brain activities in unshielded Earth's field with optically pumped atomic magnetometers. *Sci. Adv.* **6**, eaba8792 (2020).

3. G. Bison, N. Castagna, A. Hofer, P. Knowles, J.-L. Schenker, M. Kasprzak, H. Saudan, A. Weis, A room temperature 19-channel magnetic field mapping device for cardiac signals. *Appl. Phys. Lett.* **95**, 173701 (2009).

4. G. Lembke, S. N. Erné, H. Nowak, B. Menhorn, A. Pasquarelli, G. Bison, Optical multichannel room temperature magnetic field imaging system for clinical application. *Biomed. Opt. Express* **5**, 876–881 (2014).

5. S. T. Keenan, K. R. Blay, E. J. Romans, Mobile magnetic anomaly detection using a field-compensated high-T_c single layer SQUID gradiometer. *Supercond. Sci. Technol.* **24**, 085019 (2011).

6. B. A. McCullian, A. M. Thabt, B. A. Gray, A. L. Melendez, M. S. Wolf, V. L. Safonov, D. V. Pelekhov, V. P. Bhallamudi, M. R. Page, P. C. Hammel, Broadband multi-magnon relaxometry using a quantum spin sensor for high frequency ferromagnetic dynamics sensing. *Nat. Commun.* **11**, 5229 (2020).

7. I. K. Kominis, T. W. Kornack, J. C. Allred, M. V. Romalis, A subfemtotesla multichannel atomic magnetometer. *Nature* **422**, 596–599 (2003).

8. H. B. Dang, A. C. Maloof, M. V. Romalis, Ultrahigh sensitivity magnetic field and magnetization measurements with an atomic magnetometer. *Appl. Phys. Lett.* **97**, 151110 (2010).

9. V. K. Shah, R. T. Wakai, A compact, high performance atomic magnetometer for biomedical applications. *Phys. Med. Biol.* **58**, 8153–8161 (2013).

10. W. C. Griffith, S. Knappe, J. Kitching, Femtotesla atomic magnetometry in a microfabricated vapor cell. *Opt. Express* **18**, 27167–27172 (2010).

11. T. Wolf, P. Neumann, K. Nakamura, H. Sumiya, T. Ohshima, J. Isoya, J. Wrachtrup, Subpicotesla diamond magnetometry. *Phys. Rev. X* **5**, 041001 (2015).

12. C. Zhang, F. Shagieva, M. Widmann, M. Kübler, V. Vorobyov, P. Kapitanova, E. Nenasheva, R. Corkill, O. Rhrle, K. Nakamura, H. Sumiya, S. Onoda, J. Isoya, J. Wrachtrup, Diamond magnetometry and gradiometry towards subpicotesla dc field measurement. *Phys. Rev. Appl.* **15**, 064075 (2021).

13. C. L. Degen, F. Reinhard, P. Cappellaro, Quantum sensing. *Rev. Mod. Phys.* **89**, 035002 (2017).

14. J. M. Taylor, P. Cappellaro, L. Childress, L. Jiang, D. Budker, P. R. Hemmer, A. Yacoby, R. Walsworth, M. D. Lukin, High-sensitivity diamond magnetometer with nanoscale resolution. *Nat. Phys.* **4**, 810–816 (2008).

15. M. W. Doherty, V. V. Struzhkin, D. A. Simpson, L. P. McGuinness, Y. Meng, A. Stacey, T. J. Karle, R. J. Hemley, N. B. Manson, L. C. Hollenberg, S. Prawer, Electronic properties and metrology applications of the diamond NV^- center under pressure. *Phys. Rev. Lett.* **112**, 047601 (2014).

16. P. Neumann, I. Jakobi, F. Dolde, C. Burk, R. Reuter, G. Waldherr, J. Honert, T. Wolf, A. Brunner, J. H. Shim, D. Suter, H. Sumiya, J. Isoya, J. Wrachtrup, High-precision nanoscale temperature sensing using single defects in diamond. *Nano Lett.* **13**, 2738–2742 (2013).

17. J. Michl, J. Steiner, A. Denisenko, A. Bülau, A. Zimmermann, K. Nakamura, H. Sumiya, S. Onoda, P. Neumann, J. Isoya, J. Wrachtrup, Robust and accurate electric field sensing with solid state spin ensembles. *Nano Lett.* **19**, 4904–4910 (2019).

18. L. Rondin, J.-P. Tetienne, T. Hingant, J.-F. Roch, P. Maletinsky, V. Jacques, Magnetometry with nitrogen-vacancy defects in diamond. *Rep. Prog. Phys.* **77**, 056503 (2014).

19. E. Bourgeois, E. Londero, K. Buczak, J. Hruby, M. Gulka, Y. Balasubramaniam, G. Wachter, J. Stursa, K. Dobes, F. Aumayr, M. Trupke, A. Gali, M. Nesladek, Enhanced photoelectric detection of NV magnetic resonances in diamond under dual-beam excitation. *Phys. Rev. B* **95**, 041402 (2017).

20. H. Kaupp, T. Hümmer, M. Mader, B. Schlederer, J. Benedikter, P. Haeusser, H.-C. Chang, H. Fedder, T. W. Hänsch, D. Hunger, Purcell-enhanced single-photon emission from nitrogen-vacancy centers coupled to a tunable microcavity. *Phys. Rev. Appl.* **6**, 054010 (2016).

21. M. W. Doherty, N. B. Manson, P. Delaney, F. Jelezko, J. Wrachtrup, L. C. Hollenberg, The nitrogen-vacancy colour centre in diamond. *Phys. Rep.* **528**, 1–45 (2013).

22. J. Jeske, J. H. Cole, A. D. Greentree, Laser threshold magnetometry. *New J. Phys.* **18**, 013015 (2016).

23. Y. Dumeige, R. Alléaume, P. Grangier, F. Treussart, J.-F. Roch, Controling the single-diamond nitrogen-vacancy color center photoluminescence spectrum with a Fabry-Perot microcavity. *New J. Phys.* **13**, 025015 (2011).

24. S. Raman Nair, L. J. Rogers, D. J. Spence, R. P. Mildren, F. Jelezko, A. D. Greentree, T. Volz, J. Jeske, Absorptive laser threshold magnetometry: Combining visible diamond Raman lasers and nitrogen-vacancy centres. *Mater. Quantum Technol.* **1**, 025003 (2021).

25. J. L. Webb, A. F. L. Poulsen, R. Staacke, J. Meijer, K. Berg-Sørensen, U. L. Andersen, A. Huck, Laser threshold magnetometry using green-light absorption by diamond nitrogen vacancies in an external cavity laser. *Phys. Rev. A* **103**, 062603 (2021).

26. J. Jeske, D. W. M. Lau, X. Vidal, L. P. McGuinness, P. Reineck, B. C. Johnson, M. W. Doherty, J. C. McCallum, S. Onoda, F. Jelezko, T. Ohshima, T. Volz, J. H. Cole, B. C. Gibson, A. D. Greentree, Stimulated emission from nitrogen-vacancy centres in diamond. *Nat. Commun.* **8**, 14000 (2017).

27. S. R. Nair, L. J. Rogers, V. Xavier, R. P. Roberts, A. Hiroshi, O. Takeshi, Y. Takashi, A. D. Greentree, J. Jeske, T. Volz, Amplification by stimulated emission of nitrogen- vacancy centres in a diamond-loaded fibre cavity. *Nanophotonics* **9**, 4505–4518 (2020).

28. V. P. Mironov, E. A. Protasova, E. I. Lipatov, E. F. Martynovich, Generation of laser radiation by color centers in diamond crystals (review). *AIP Conf. Proc.* **2392**, 030001 (2021).

29. E. Fraczek, V. G. Savitski, M. Dale, B. G. Breeze, P. Diggle, M. Markham, A. Bennett, H. Dhillon, M. E. Newton, A. J. Kemp, Laser spectroscopy of NV- and NV0 colour centres in synthetic diamond. *Opt. Mater. Express* **7**, 2571 (2017).

30. A. Savvin, A. Dormidonov, E. Smetanina, V. Mitrokhin, E. Lipatov, D. Genin, S. Potanin, A. Yelisseyev, V. Vins, NV- diamond laser. *Nat. Commun.* **12**, 7118 (2021).

31. V. G. Savitski, Optical gain in NV-colour centres for highly-sensitive magnetometry: A theoretical study. *J. Phys. D Appl. Phys.* **50**, 475602 (2017).

32. L. Hacquebard, L. Childress, Charge-state dynamics during excitation and depletion of the nitrogen-vacancy center in diamond. *Phys. Rev. A* **97**, 063408 (2018).

33. Y. Dumeige, J.-F. Roch, F. Bretenaker, T. Debuisschert, V. Acosta, C. Becher, G. Chatzidrosos, A. Wickenbrock, L. Bougas, A. Wilzewski, D. Budker, Infrared laser threshold magnetometry with a NV doped diamond intracavity etalon. *Opt. Express* **27**, 1706–1717 (2019).

34. R. Albrecht, A. Bommer, C. Deutsch, J. Reichel, C. Becher, Coupling of a single nitrogen-vacancy center in diamond to a fiber-based microcavity. *Phys. Rev. Lett.* **110**, 243602 (2013).

35. C.-H. Su, A. D. Greentree, L. C. L. Hollenberg, Towards a picosecond transform-limited nitrogen-vacancy based single photon source. *Opt. Express* **16**, 6240 (2008), 6250.

36. M. Pollnau, M. Eichhorn, Spectral coherence, Part I: Passive-resonator linewidth, fundamental laser linewidth, and Schawlow-Townes approximation. *Prog. Quantum Electron.* **72**, 100255 (2020).

37. A. Siegman, *Lasers* (University Science Books, 1986).

38. P. W. Milonni, J. H. Eberly, *Laser Physics* (John Wiley & Sons, 2010).

39. A. Dréau, M. Lesik, L. Rondin, P. Spinicelli, O. Arcizet, J.-F. Roch, V. Jacques, Avoiding power broadening in optically detected magnetic resonance of single NV defects for enhanced dc magnetic field sensitivity. *Phys. Rev. B* **84**, 195204 (2011).

40. M. Capelli, "Investigation of artifcial diamonds for optical sensing with ensemble of nitrogen-vacancy centres," thesis, RMIT University (2019).

41. J. F. Barry, J. M. Schloss, E. Bauch, M. J. Turner, C. A. Hart, L. M. Pham, R. L. Walsworth, Sensitivity optimization for NV-diamond magnetometry. *Rev. Mod. Phys.* **92**, 015004 (2020).

42. R. Staacke, R. John, R. Wunderlich, L. Horsthemke, W. Knolle, C. Laube, P. Glösekötter, B. Burchard, B. Abel, J. Meijer, Isotropic scalar quantum sensing of magnetic fields for industrial application. *Adv. Quantum Technol.* **3**, 2000037 (2020).

43. P. Kehayias, M. W. Doherty, D. English, R. Fischer, A. Jarmola, K. Jensen, N. Leefer, P. Hemmer, N. B. Manson, D. Budker, Infrared absorption band and vibronic structure of the nitrogen-vacancy center in diamond. *Phys. Rev. B* **88**, 165202 (2013).

44. A. T. Collins, Optical centres produced in diamond by radiation damage, *New Diam. Front. Carbon Technol.* **17**, 47 (2007).

45. C. Cohen-Tannoudji, B. Diu, F. Laloe, *Quantum Mechanics, Volume 1: Basic Concepts, Tools, and Applications* (Wiley-VCH, 2020).
